# Supplementary material for: A human cadaveric model for venous air embolism detection tool development
Source: PLoS One. 2024 Oct 21;19(10):e0309447. doi: 10.1371/journal.pone.0309447 (PMC11493288; doi:10.1371/journal.pone.0309447)
Supplement: Attachment — Submitted filename: Response to Reviewers.docx [file pone.0309447.s003.docx]

Priti Chaudhary, M.S.

Editor-in-Chief, *PLoS ONE*

Dear Editor,

We are grateful for the insightful comments of the reviewers. In response to their feedback and editorial requirements, we have edited the manuscript using tracked changes, and describe those changes below with our responses to the authors’ queries. The requests made by the editors have also been made as well.

Thank you,

Chris Marcellino, M.D.

**Reviewer #1:**

**Reviewer #1: The introduction is well constructed highlighting the aim of the work, addition of some statistics regarding prevalence of VAE during surgeries, incidence of resulting disabilities and complication is a plus. The methodology detailed description of the three approaches to establish a simulation method and minimize fluid loss.**

**The results: are the recordings done in several sessions to the conducted model??!!! if not several recordings in different trials for the finalized intrathoracic approach is a plus with comparison between the single pair of electrodes to multiple ones.**

Yes, the recordings were done in one day on the same cadaver, due to limitations in cadaveric access and the costs of disposable equipment used in cardiac bypass. We progressively extended the dissection as described and repeated extracorporeal circulation until we hit the limitations of volume overload.

**The discussion clearly enlisted the limitations of the study with recommendation of further studies to validate the model. It needs enrichment with other studies e.g. reviews supporting using colloids instead of saline to support intravascular volume expansion.**

Thank you for raising that point. We have added 4 citations contained in the additional discussion on page 9.

**Reviewer #2: The manuscript is technically sound and well written. It will help in further understanding of techniques in detection of venous air emboli especially in vivo human. However, find below some minor corrects and suggestions for possible improvement in the quality of the manuscripts.**

**The word aorta was used in significant component of the manuscript, it is good specify the part of aorta use in each context e.g. abdominal aorta, descending thoracic aorta etc.**

Thank you for identifying this omission. This was corrected in the revised manuscript (though the reference in the abstract was left intentionally ambiguous given that it refers to multiple sites.)

**“Small intestine” is the more scientifically precise term, while “small bowel” is commonly used in clinical settings. But “small intestine” is generally preferred in scientific and medical literature. The use of words normal saline is more preferable than the word saline.**

Thank you. All occurrences of both of these errors were corrected.

**The SVC was mentioned before that of Line 154 SVC. Please abbreviate the SVC in the first instance.**

Thank you for your diligence in editing the manuscript. It appears the first reference of superior vena cava is on Page 7, what is now line 188, and it is only used thereafter in the same paragraph in abbreviation (line 195 & 196), so no changes were made. IVC is also verified to corrected expanded and abbreviated. Thank you again for verifying this.

**Line 181 the… example.) should be example). The full stop should be after the bracket.**

This was corrected. Thank you for noting this.

**Lines 194 other experimenters… are you referring to more than one study? If yes. Please provide additional references.**

Thank you. I have added a clarifying comment to page 8, line 237 to reflect that we only found this single study. None of our colleagues are aware of other studies and we could not find any others via PubMed or Google.

**In my opinion, the use of one cadaver to develop a model may not be adequate. So this issue of utilizing only one cadaver should discussed in the manuscripts as part of the limitations or recommended for further exploration.**

Yes, this is a main weakness of this manuscript. We have added a passage to the end of the penultimate paragraph of the manuscript on page 9 to reflect this. It is clear we have not quantitatively proven that this is an effective platform for research, but instead we seek to qualitatively share a possible scheme or a starting point for cadaveric experimentation into the public domain for others to use if they see fit, especially given the paucity of alternatives in the literature (as I mentioned above.)

In the interval since we have authored this manuscript, we have been working to develop minimally invasive tools for air entrainment monitoring, and hope to one day have a validated device which can be either shared academically and perhaps commercialized as well. This, however, may be a long time ahead and may not feasible at all. We hope others may continue the same research as we feel that VAE is an underserved and understudied area of perioperative morbidity. Thank you again for your time spent reviewing this manuscript.
